# Magnetic Reference Mark in a Linear Positioning System Generated by a Single Wiegand Pulse

**DOI:** 10.3390/s22093185

**Published:** 2022-04-21

**Authors:** Hung-Lin Lien, Jen-Yuan Chang

**Affiliations:** Department of Power Mechanical Engineering, National Tsing Hua University, Hsinchu 30013, Taiwan; a0903553298@gmail.com

**Keywords:** Wiegand effect, Wiegand sensor, Wiegand pulse, reference mark

## Abstract

A Wiegand sensor is composed of a strip of Wiegand wire and a pick-up coil. The research presented in this paper examines and characterizes the fast magnetization reversal in a Wiegand wire, which leads to changes in magnetic flux density in its pick-up coil to produce the so-called Wiegand pulse to be used as a reference mark in a linear positioning system. It was observed in this research that the magnitude and duration of the pulse voltage were independent of driving frequency, indicating that Wiegand effect sensors could be ideal for use as zero-speed transducers. The repeatability of the Wiegand pulse was found to vary with different magnetic flux intensities of external magnetic field, as well as the angle between the magnetic induction line and the Wiegand wire. Through calibrated experimental and numerical parametric studies, the mechanism for producing repeatable Wiegand pulses to be used as a reference mark for precision liner positioning systems was revealed, which represents the novelty of this research. On the basis of this mechanism, the optimal design combination of the Wiegand sensor’s position with respect to the magnetization source can be obtained. Utilizing commercially available Wiegand sensors, it was demonstrated in this research that with a Wiegand pulse serving as a magnetic reference mark, positioning repeatability of 0.3 um could be achieved, which is on the same order as optical scales. The work presented in this research has engineering implications as well as offering scientific insights into magnetization mechanisms for generating enough magnetic remanence to produce a Barkhausen jump, resulting in repeatable Wiegand for use as a reference mark in a linear positioning system.

## 1. Introduction

With industry’s requirement for precision, linear positioning systems have been widely used in various applications, such as machine tools, laser processing, and robotic arms. With the increasing use of linear positioning systems for higher precision, there is an increasing demand for linear positioning systems with higher accuracy. Because of the kinematic errors and straightness errors in the linear motor stage, the degradation of precision accuracy is commonly observed. The commonly used method for improving accuracy is by using laser interferometers to measure the positioning errors, on the basis of which a corresponding error map can be built. The error map is then stored in the motion controller to compensate the positioning error in real time. Since this method requires a reference mark as the starting point of the pre-built error map, the reference mark inevitably requires high repeatability so that the error map can correctly and effectively compensate for the positioning error.

There are three main types of reference mark: one is a precision mechanical switch, another is an optical reference mark, and the other is a magnetic reference mark. The precision mechanical switch offers the lowest cost, but it has the problem of mechanical wear. The optical reference mark has the advantage of no mechanical wear, and it has the highest repeatability and resolution among the three mentioned types of reference mark. However, the optical reference mark cannot maintain its performance under severe ambient conditions caused by oil, water, or dust, for example. The magnetic reference mark, on the other hand, is able to solve this problem. It can be used under severe ambient conditions with no mechanical wear, and also achieve high repeatability. Based on the advantages of the magnetic reference mark and the Wiegand sensor, a single Wiegand pulse was applied and examined for its potential to be used as a reference mark in a linear position system in this research. Specifically, the relationship between the Wiegand pulse’s peak voltage and different magnetic circuits was investigated and examined. Calibrated experimental and analytical studies were conducted to examine the variation and sensitivity of the pulse’s repeatability in different magnetic circuits. An appropriate configuration of magnets used to generate the Wiegand pulse was developed this research, so as to optimize the repeatability of the Wiegand pulse for use as the reference mark for precision linear positioning systems.

Although extensive research has been reported in the literature using the Wiegand effect for energy harvesting and counting applications, such as that for tachometers in rotating machines, not much has, like this research, addressed using the Wiegand pulse to serve as a magnetic reference mark for a linear positioning system. The proposed idea of the Wiegand pulse as a magnetic reference mark is original and innovative. The magnetization mechanism that will be presented in the following content of this paper to produce a repeatable Wiegand pulse with a repeatability of 0.3 um (same level as optical scales) is thought to have scientific archival value that could contribute to the body of applied research as well as to the design of magnetic reference marks for precision linear positioning systems in smart machine applications.

## 2. Wiegand Sensor

### 2.1. Wiegand Effect

**Figure 1:**
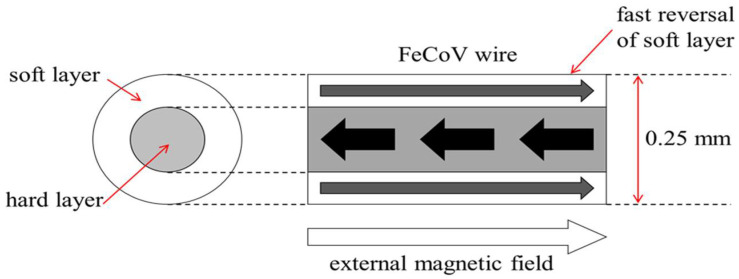
Schematic drawing of magnetic structures of the FeCoV wire.

**Figure 2:**
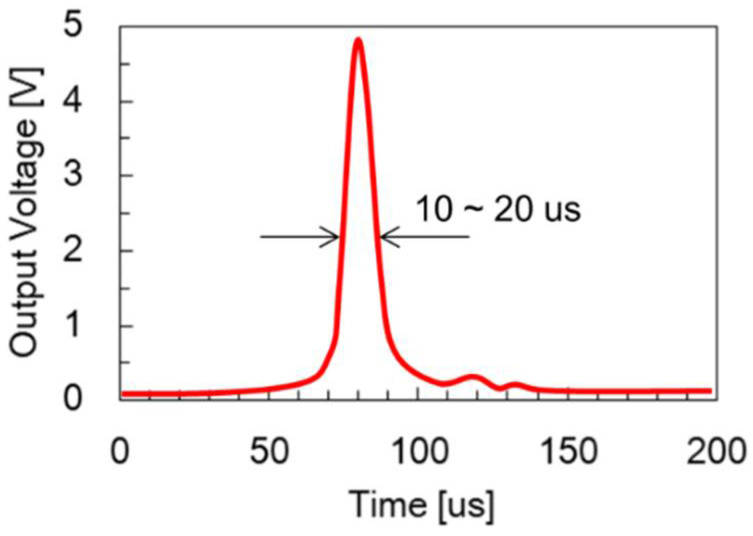
Waveform of pulse voltage obtained from a Wiegand sensor.

The Wiegand effect was initially observed in NiFe alloy wire by John R. Wiegand in 1974 [1,2]. It was reported that the wire made of FeCoV alloy offered optimum performance in generating the Wiegand effect [3,4,5,6,7,8,9]. The diameter of the Wiegand wire used in the prior research was about 0.25 mm. Heat treatment such as quenching and annealing were applied to the wire in order to change the alloy’s grain size. After heat treatment, cold treatment such as stretching and twisting were then introduced to the wire to cause it to display different degrees of coercivity in its surface region and center region, respectively. Figure 1 shows the “hard layer”, with relatively high coercivity, and the “soft layer”, with relatively low coercivity, in FeCoV alloy wire after the aforementioned treatments. When the intensity of the external magnetic field passing through the axial direction of the wire with different coercivities reaches a certain threshold, the “soft layer” with relatively low coercivity will fast reverse its direction of magnetization. The fast magnetization reversal of the soft layer leads to the generation of a large Barkhausen jump, resulting in changes of magnetic flux in the pick-up coil, such that a sharp pulse with a duration of roughly 10~20 µs can be induced [10]. Figure 2 shows the waveform of the pulse voltage obtained from a Wiegand sensor. This pulse voltage is also called a Wiegand pulse.

**Figure 3:**
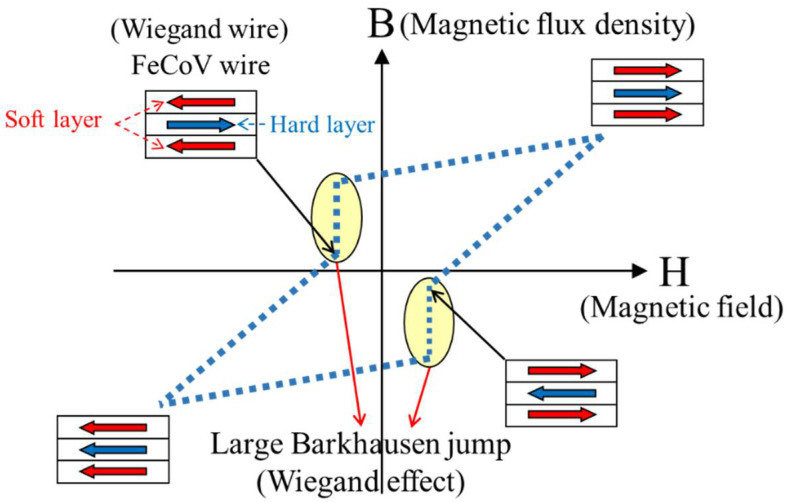
Schematic illustration of Barkhausen jump due to fast magnetization reversal between soft and hard layers of a Wiegand wire.

The effect of using an alloy wire with two coercivities whereby the fast magnetization reversal of the wire’s soft layer can repeatedly induce a large Barkhausen jump as a result of the external magnetic field is called the Wiegand effect. Figure 3 shows an exemplary B-H curve obtained from a Wiegand wire when subjected to an external magnetic field H, where B stands for the magnetic flux density within the Wiegand wire. When generating the Wiegand pulse, the external magnetic field H, with appropriately high strength, should be applied and pass through the wire’s axial axis to magnetize both soft and hard layers of the Wiegand wire. With such strong magnetization, the magnetic orientation of materials in the soft and hard layers are in the so-called parallel state, because they are forced to orient to the same magnetic orientation, as illustrated by the red and blue arrows in the first quadrant of the figure. When the external magnetic field is removed, leading to zero value in H, both layers of the Wiegand wire possess the same magnetic remanence (Br). As shown in the second quadrant of the figure, when the opposite direction of the external magnetic field is applied to the Wiegand wire, the wire’s soft layer responds faster than the hard layer to the negative value of H, resulting in reversal of its magnetic polarity. This is commonly referred to as the anti-parallel state in the literature. The difference in the magnetic properties between the soft and hard layer of the material in response to the external magnetic field reversal is in fact the mechanism that results in the magnetic orientation reversal generating the Wiegand pulse. When the strength of the reversed external magnetic field increases, as shown in the third quadrant of the figure, the magnetic orientations of the soft and hard layers are then once again forced to be aligned in the same direction, but in the opposite direction to that in the first quadrant. When this reversed external magnetic field is gradually removed, both layers possess the same negative remanence, and the phenomenon repeats. It should be noted that to be able to create the Wiegand effect or the so-called Barkhausen jump as indicated in Figure 3, enough remanence value for both layers is a prerequisite, and the degree of difference between the soft and hard layer materials when responding to the external magnetic reversal can determine the waveform pulse voltage obtained from the Wiegand sensor. These two factors are evidently related to the design of the external magnetic field relative to the Wiegand wire, which will be investigated, examined, and discussed in context in this paper.

### 2.2. Applications

The magnitude and width of the pulse has been reported to be independent of driving frequency. This phenomenon could be used as a zero-speed sensor. Reported in [11], the Wiegand sensor was able to generate electrical power of 600 nJ, which could be used for driving low-power electronic devices. The material of the Wiegand sensor is stable and cheap, and the generated pulse energy does not decay after switching its magnetic polarities a million times. Advantaged by the above features, Wiegand sensors have been used in energy harvesting [12], rotary speed transducers [13], and multi-turn absolute encoders [14].

Recently, energy harvesting for the Internet of Things (IoT) has attracted a great deal of attention [15,16,17]. In installing electronic devices, it is difficult to use power cables to provide power. Although devices with batteries may solve this problem, the batteries inevitably need to be replaced after a certain period of time. This problem can be effectively solved by using self-generating sensors as power supplies. In this study, we applied the Wiegand sensor to a linear positioning system, not only so that the Wiegand pulse could be used as a reference mark, but also because it does not require an additional power supply. With the advantage of battery-less operation, the focus of this paper is then placed on the design of the system’s magnetic circuits so that by adjusting the position of the Wiegand sensor, the Wiegand pulse can be effectively triggered to reach high repeatability.

## 3. Methods

**Figure 4:**
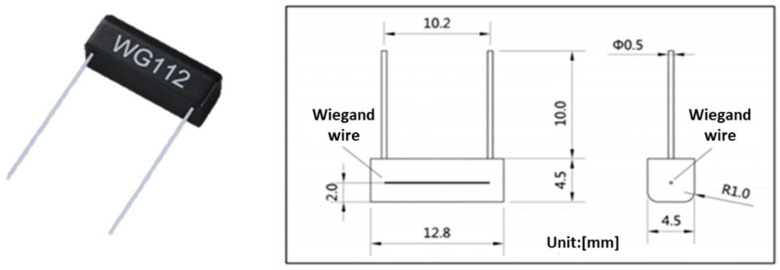
Dimensions of the commercial WG112 Wiegand sensor used in this research.

Commercial Wiegand sensor WG112 manufactured by Nanjing AH Electronic Science & Technology was employed in this research. The dimensions of the WG112 Wiegand sensor are shown in Figure 4.

### 3.1. Linear Measuring System

**Figure 5:**
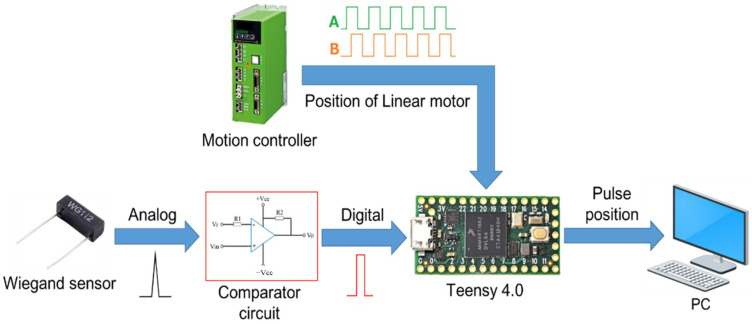
Process of measuring repeatability of the Wiegand pulse.

**Figure 6:**
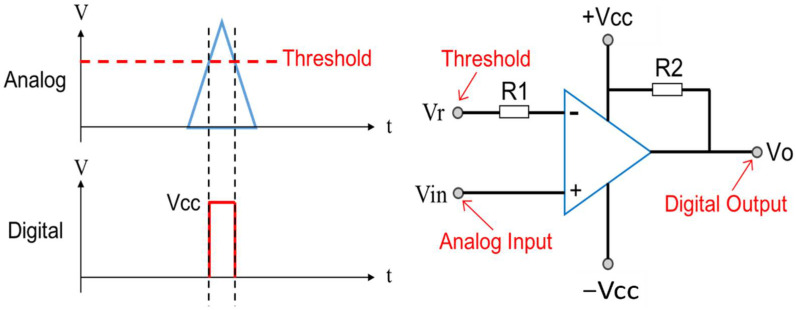
Schematic drawing of the comparator circuit.

To investigate the feasibility of using the Wiegand pulse as a refence mark, a linear ironless motor HIWIN LMCB5 equipped with 0.1-um-resolution optical encoder, namely, RENISHAW RGH22 was employed. Permanent magnets were carried by the moving part of the linear motor, whereas the Wiegand sensor was installed in the inertia reference frame. Figure 5 shows the process of measuring the repeatability of the Wiegand pulse. The analog Wiegand pulse is first inputted into the comparator circuit as shown in Figure 6, from which circuit the analog Wiegand pulse signal is digitized. The threshold of the comparator circuit is set to 0.25 V so that the signal is not easily affected by ambient noise, where the signal level of the noise is about 0.1 V. The A and B orthogonal signals generated by the optical encoder are then inputted into the Teensy 4.0 microcontroller, both of which signals are synchronized with the input of the digitized Wiegand pulse signal. With this process, as the Wiegand pulse occurs, the microcontroller can then output the position of the magnets on the moving part of the linear motor-based upon the pulse generated from the Wiegand sensor to the computer. This research investigated the configuration of the magnets and the relevant position and orientation of the Wiegand sensor to generate repeatable Wiegand pulse.

### 3.2. Position Adjustment of the Wiegand Sensor

**Figure 7:**
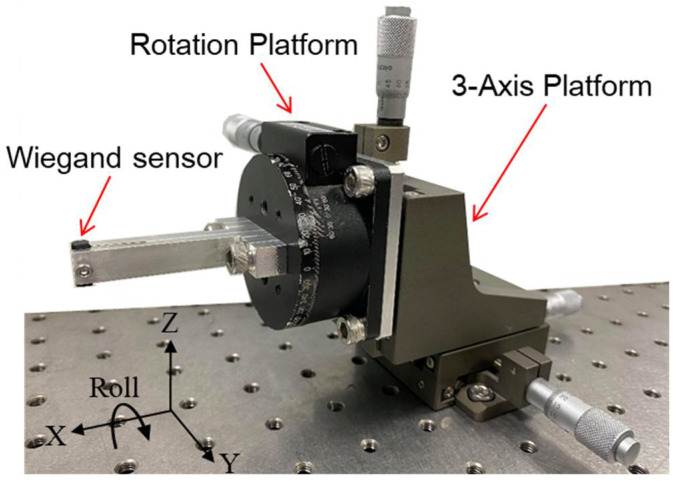
Precision displacement platform carrying the Wiegand sensor.

**Figure 8:**
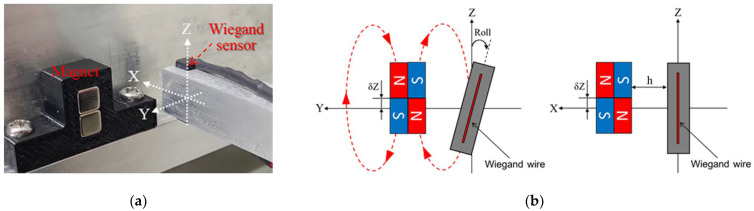
Configuration of Wiegand sensor and magnets: (**a**) isometric view (**b**) front view and side view.

As shown in Figure 7, the Wiegand sensor is installed at the tip of a rigid rectangular beam that is attached to the precision displacement platform to adjust its X, Y, and Z displacements, as well as its roll angular displacement with respect to the linear motor. The displacement platform is fixed in the inertia reference frame, the same as the linear motor. Based on the Wiegand effect reported in the literature, two N35 (NdFeB) permanent magnets forming a magnet pair were adopted to produce external magnetic field to the Wiegand sensor. The size of each of the magnets is 5 × 5 × 5 mm. The magnetic polarity was either north, N, or south, S, around each end of the magnets, with the ends being opposite to each other. The placements of the Wiegand sensor and the arrangement of magnetic polarity of the two magnets are shown in Figure 8a,b, respectively.

**Figure 9:**
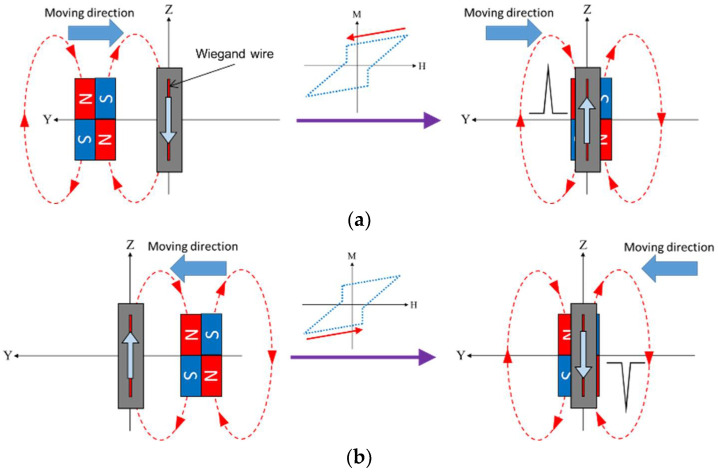
Schematic illustration of the process of pulse signal generation as the magnet pair is moved along the Y-axis from (**a**) left to the right and (**b**) right to the left.

The linear motor carrying the magnet was set to move back and forth along the Y-axis so as to trigger the Wiegand pulse. Figure 9 gives the schematic illustration of the generation of the pulse signal. When the magnet pair was far from the Wiegand sensor, the magnetizations of the soft and hard layers of the Wiegand wire were the same. It can be observed that positive and negative pulses were generated because the wire’s soft layer rapidly reversed to opposite direction, and the pulses occurred, from which the center of the magnet pair moved to about Y = ±0.1 mm. To obtain the relationship between the angle and the position of the Wiegand sensor and the repeatability of the Wiegand pulse with the aforementioned linear measuring system, h, the distance between the Wiegand wire and the magnets, was set to 0.5, 1.0, and 1.5 mm, the Roll angle was set to −4, −3, −2, −1, 0, 1, 2, 3, and 4 degrees, and δZ, the vertical offset of the geometrical center of the magnet pair and the Wiegand wire, was set to −1.5, −1.0, −0.5, 0, 0.5, 1.0, and 1.5 mm, respectively, in the numerical and experimental parameter studies presented below.

### 3.3. Distribution of the Magnetic Field

**Figure 10:**
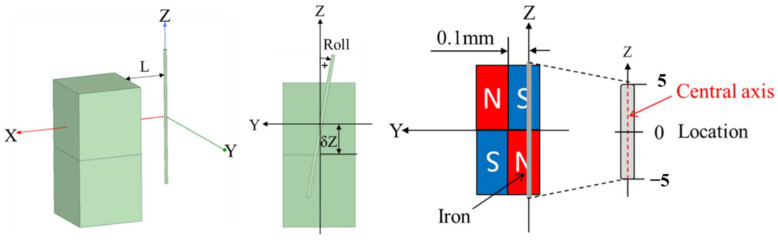
Configuration of Wiegand wire and magnet pair in simulation.

Before the experiments, the magnetic flux density through the Wiegand wire in the radial and axial axes were simulated by finite element analysis using ANSYS Maxwell^®^ (ANSYS, Inc., Canonsburg, PA, USA). In the simulation, as shown in Figure 10, the 3D models of the Wiegand wire and the magnet pair were defined. The corresponding material of the magnet was set as NdFeB35, and the Wiegand wire was designed as a cylinder with a radius of 0.125 mm and a height of 10 mm. To simulate the magnetization of the wire’s soft layer as the Wiegand pulse occurred, the position of the magnets’ center was set to Y = −0.1, 0.1 mm from the Wiegand wire, and the material of Wiegand wire was set to iron, which was the same as the soft magnetic material. In order to analyze the influence of Roll angle and δZ offset on the magnetic flux density through the Wiegand wire, h was first set to 1.0 mm in the experiments. In consideration of a package height of 2 mm in the WG112 Wiegand sensor, the distance from the surface of the magnet pair to the Wiegand wire should be set to L = 3.0 mm to account for the packaging effect. Roll angle and δZ offset were set to −2, 0, and 2 degrees and −2, 0, and 2 mm, respectively, to analyze the distribution of the magnetic flux density through the Wiegand wire.

**Figure 11:**
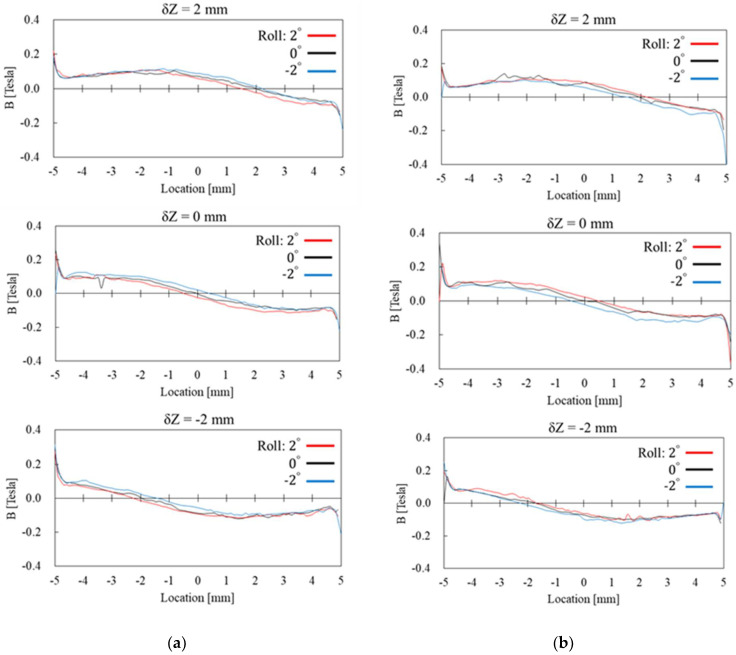
Simulation results of the radial magnetic flux density through the Wiegand wire with Y being set at (**a**) −0.1 mm and (**b**) 0.1 mm, respectively.

**Figure 12:**
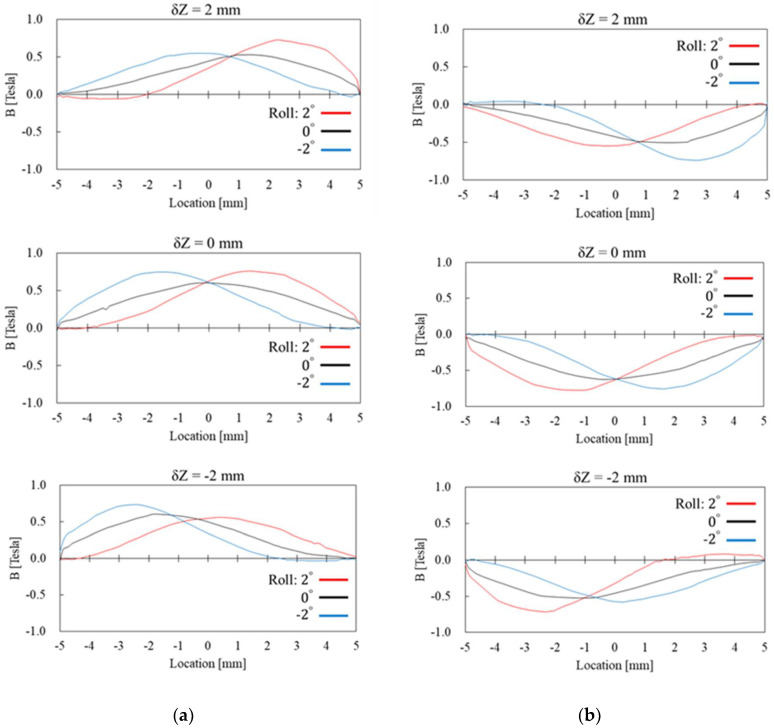
Simulation results of the axial magnetic flux density through the Wiegand wire with Y being set at (**a**) −0.1 mm and (**b**) 0.1 mm, respectively.

The simulation results presented in Figure 11 and Figure 12 are the radial and axial components of magnetic flux density through the Wiegand wire, respectively. The “Location” in the horizontal axis of those figures indicates the location along the Z axis of the Wiegand wire, as shown in Figure 10. Cases of Y = −0.1 mm and Y = 0.1 mm, corresponding to Figure 9a,b, are the conditions when positive and negative Wiegand pulses are generated, respectively. It can be observed that non-zero δZ shifts the near-symmetric distribution of the radial magnetic flux density with respect to Location = 0 mm, whereas the Roll angle has low sensitivity to the distribution of the radial magnetic flux density. However, the Roll angle has high sensitivity to the distribution of the axial magnetic flux density. A phenomenon is observed whereby if the distributions of axial magnetic flux density in positive and negative pulses correspond with one another, the angle of Roll should be the opposite number. For example, in Figure 12, when δZ = 2 mm, if Roll = 2 degrees for the positive pulse (the red curve in the figure), the corresponding distribution of the axial magnetic flux density for the negative pulse will be Roll = −2 degrees (the blue curve in the figure).

**Figure 13:**
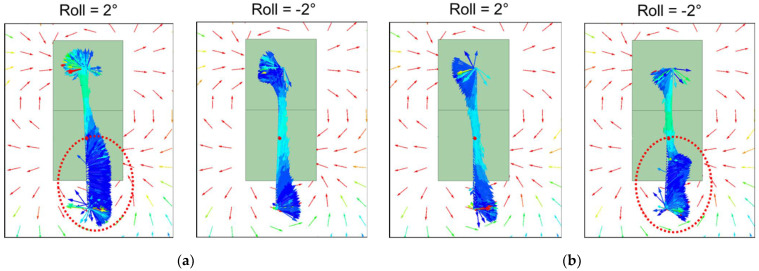
Simulation results of the magnetization direction of the Wiegand wire as indicated by colored arrows when δZ = 2 mm with Y being set at (**a**) −0.1 mm and (**b**) 0.1 mm, respectively. The red-circled regions highlight region of the wire when ratio of the magnetization direction in the radial axis increases due to the wire’s soft layer suddenly reverses its polarity.

The simulation results in Figure 12 also show that if δZ increases or decreases with specific angle directions of Roll, the distribution of the axial magnetic flux density can display a large shift from Location = 0 mm, the axial center of the Wiegand wire. Using δZ = 2 mm and Roll = 2 degrees as an example, there is a large shift in the distribution of the axial magnetic flux density when Y = −0.1 mm, and the magnetic flux density in the axial direction is close to 0 Tesla at Location = −2~−5 mm. Therefore, the ratio of the magnetization direction in the radial axis increases when the wire’s soft layer suddenly reverses its polarity, as shown in the red-circled region in Figure 13. On the other hand, the ratio of the magnetization direction in the radial axis can be controlled by adjusting values of Roll angle and δZ offset.

## 4. Results and Discussion

### 4.1. Relationship between the Repeatability and the Position of the Wiegand Sensor

**Figure 14:**
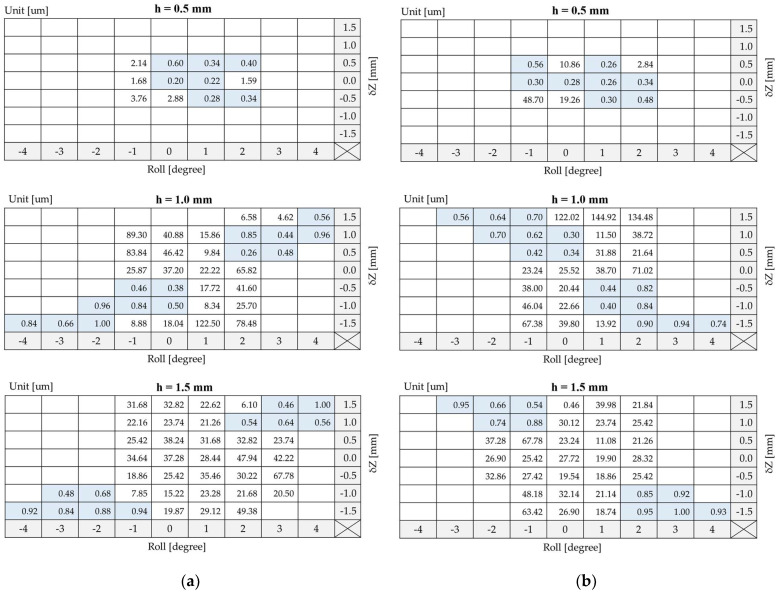
Experimental results of the standard deviations of (**a**) positive pulse and (**b**) negative pulse generated by the Wiegand sensor with different Roll angle and δZ offset values when h = 0.5, 1.0, 1.5 mm. The blue boxes highlight results of high position repeatability less than 1 um.

Figure 14 shows the experimental results of the standard deviations of the reference mark position judged by the occurrence of the Wiegand pulse from the linear measuring system with different Roll angle and δZ offset values when h = 0.5, 1.0, 1.5 mm. Each standard deviation was generated by computing every 100 runs of measurements. It can be observed that when the Roll and δZ and h are adjusted to appropriate values, the standard deviations can be less than 1 um, indicating the high repeatability of using the Wiegand pulse as a reference mark in linear positioning systems. From the checkerboard tables in Figure 14, it can be observed that when h varies between 0.5 mm and 1.5 mm, the standard deviations for both positive and negative Wiegand pulses less than 1 um (highlighted by light blue shaded blocks) are distributed from the middle to the diagonal. The distribution of the standard deviations in the checkerboard tables for the positive and negative pulses are not symmetrical with each other. Determined by the standard deviation values when h = 1.0 mm and δZ = 1 mm, the repeatability of the positive pulse can be improved by adjusting the Wiegand sensor’s Roll angle in the positive direction from zero degrees. However, for the negative pulse, its repeatability cannot be improved by adjusting the Roll angle value in the positive direction, but rather in the negative direction.

### 4.2. Relationship between the Repeatability and Magnetic Flux Density

Through the calibrated experiments described above, the relationship between the repeatability and the position of the Wiegand sensor as a reference mark was determined. By comparing experiments to simulations, it was observed that when the wire’s soft layer suddenly reverses its polarity, the ratio of the magnetization direction in the radial axis increases, leading to improvement in the repeatability of the reference mark position determined by the Wiegand pulse. For example, as shown in Figure 14a for a positive pulse, if δZ is a positive value, the pulse’s repeatability can be improved by adjusting the angle of Roll in a positive direction. Compared to the simulation, as shown in Figure 12 and Figure 13, when δZ is a positive value, if the angle of Roll is adjusted in the positive direction, the distribution of the axial magnetic flux density will be greatly shifted, thereby increasing the ratio of the magnetization direction in the radial axis. For negative pulses, if δZ is a positive value, the angle of Roll should be adjusted in the negative direction, which is opposite to what was just described for positive pulses, to improve the pulse’s repeatability.

**Figure 15:**
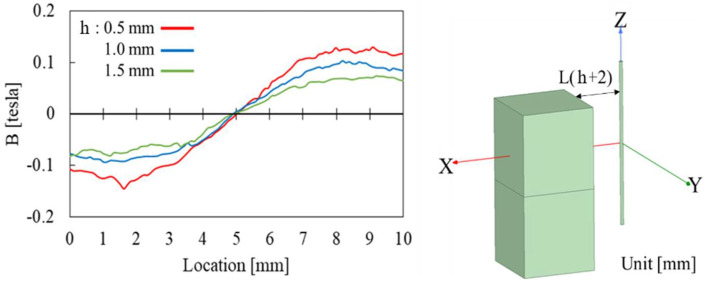
Simulation results of the magnetic flux density through the Wiegand wire in the radial axis when h = 0.5, 1.0, 1.5 mm.

By comparing the variation of magnetic flux density in the radial axis as shown in Figure 15, the magnitude of radial magnetic flux density through the Wiegand wire was found to increases with decreasing h. As a result, when h = 0.5 mm, the radial magnetic flux density in the Wiegand wire is strong enough that the high repeatability of the Wiegand pulses is distributed in the center region of the checkerboard table, as shown in Figure 14. If one were to adjust the δZ and Roll values too much, the ratio of the magnetization direction in the radial axis would be too large, reducing the effective magnetic field applied to the Wiegand wire, thereby decreasing the peak voltage. As the result, the pulse may be too small to be detected. Therefore, when h varies between 0.5 mm and 1.5 mm, the standard deviations of the Wiegand pulse, which are less than 1 um, are distributed from the middle to the diagonal region in the checkerboard tables.

This trend between simulations and experiments as described above is found to be consistent. Additionally, it was observed that the experimental results exhibited a high correlation to the simulation results. It can be concluded that there are two methods for increasing the ratio of the magnetization direction in the radial axis, leading to improvements in the repeatability of the reference mark, and one of those methods is to adjust the Roll angle and δZ offset to an appropriate value, while the other method is to decrease the flying height h.

## 5. Conclusions

In this paper, a novel magnetic reference mark utilizing a single Wiegand pulse for linear positioning systems was proposed, examined, and validated. Through a large number of experimental and numerical parameter studies, the key factors for producing and even improving the repeatability of the Wiegand pulse were determined. The magnetization mechanism and relationship between magnetization source and the magnetic flux density through the Wiegand wire required to produce repeatable Wiegand pulse were revealed in this work. On the basis of this study, it is suggested that when using the Wiegand pulse as a reference mark, a fine adjustment device should be applied to adjust δZ offset and Roll angle values of the Wiegand wire with respect to the magnetization source, such as magnet pairs, to appropriate parameters in order to obtain high positioning repeatability. It was found in this research that the optimal standard deviations of the Wiegand pulse were less than 0.3 um, which is on the order of values produced by optical scales. As Wiegand sensors out-perform optical sensors under severe ambient conditions caused by oil or water, for example, the work presented in this study has engineering implications in that a magnetic reference mark using a Wiegand pulse has the potential to be a substitute for optical reference marks, particularly for precision machines in factories requiring positioning control with compensation for errors in order to increase their accuracy. The research results reported in this paper not only reveal and validate the fact that the ratio of the magnetization direction in the Wiegand wire’s radial axis is the key to improving its repeatability, they also offer design guidelines regarding the placement of the magnet pair and the Wiegand sensor in a linear positioning system, as well as the assembly specifications of the δZ offset and Roll angle of the Wiegand sensor required to generate high position repeatability as a reference mark. The scientific contribution of the work presented in this paper is related to our understanding of the magnetization mechanism for producing sufficient magnetic remanence in soft and hard layers of Wiegand wire offering large Barkhausen jumps to produce repeatable Wiegand pulses for use as a magnetic reference mark, which is of scientific archival value for engineers in designing magnetic reference marks for positioning control in smart machines.

## Data Availability

Not applicable.
